# Chimeric Antigen Receptor T-Cells for the Treatment of B-Cell Acute Lymphoblastic Leukemia

**DOI:** 10.3389/fimmu.2018.00239

**Published:** 2018-02-19

**Authors:** Ciprian Tomuleasa, Shigeo Fuji, Cristian Berce, Anca Onaciu, Sergiu Chira, Bobe Petrushev, Wilhelm-Thomas Micu, Vlad Moisoiu, Ciprian Osan, Catalin Constantinescu, Sergiu Pasca, Ancuta Jurj, Laura Pop, Ioana Berindan-Neagoe, Delia Dima, Shigehisa Kitano

**Affiliations:** ^1^Department of Hematology, Oncology Institute Prof. Dr. Ion Chiricuta, Cluj Napoca, Romania; ^2^Research Center for Functional Genomics and Translational Medicine, Department of Hematology, Iuliu Hatieganu University of Medicine and Pharmacy, Cluj Napoca, Romania; ^3^Department of Stem Cell Transplantation, Osaka International Cancer Institute, Osaka, Japan; ^4^Animal Facility, Iuliu Hatieganu University of Medicine and Pharmacy, Cluj Napoca, Romania; ^5^Research Center for Functional Genomics and Translational Medicine, Iuliu Hatieganu University of Medicine and Pharmacy, Cluj Napoca, Romania; ^6^Department of Hematology, Iuliu Hatieganu University of Medicine and Pharmacy, Cluj Napoca, Romania; ^7^Division of Cancer Immunotherapy, Department of Experimental Therapeutics, National Cancer Center Hospital, Tokyo, Japan

**Keywords:** acute lymphoblastic leukemia, immunotherapy, chimeric antigen receptor T-cell therapy (CAR-T), gene transferred T-cell therapy, adoptive cell transfer

## Abstract

Chimeric antigen receptor (CAR) T-cell technology has seen a rapid development over the last decade mostly due to the potential that these cells may have in treating malignant diseases. It is a generally accepted principle that very few therapeutic compounds deliver a clinical response without treatment-related toxicity, and studies have shown that CAR T-cells are not an exception to this rule. While large multinational drug companies are currently investigating the potential role of CAR T-cells in hematological oncology, the potential of such cellular therapies are being recognized worldwide as they are expected to expand in the patient to support the establishment of the immune memory, provide a continuous surveillance to prevent and/or treat a relapse, and keep the targeted malignant cell subpopulation in check. In this article, we present the possible advantages of using CAR T-cells in treating acute lymphoblastic leukemia, presenting the technology and the current knowledge in their preclinical and early clinical trial use. Thus, this article first presents the main present-day knowledge on the standard of care for acute lymphoblastic leukemia. Afterward, current knowledge is presented about the use of CAR T-cells in cancer immunotherapy, describing their design, the molecular constructs, and the preclinical data on murine models to properly explain the background for their clinical use. Last, but certainly not least, this article presents the use of CAR T-cells for the immunotherapy of B-cell acute lymphoblastic leukemia, describing both their potential clinical advantages and the possible side effects.

## Current Management of Acute Lymphoblastic Leukemia (ALL)

Acute leukemias are classified into acute myeloid leukemia (AML) and ALL, depending on the result of immunophenotype characterization. The latest World Health Organization classification replaced the classic cytological classification of ALLs into B-cell ALL and T-cell ALL. B-cell ALL and acute lymphoblastic lymphomas are malignancies with B-cell lymphoblasts (1–3). In B-cell ALLs, the bone marrow aspirate will detect at least 20% bone marrow lymphoblasts, with this type of malignancies representing around 85% of all pediatric ALLs (4–6). The accurate diagnosis is made after flow cytometry immunophenotyping, with positive cells for CD10, CD19, CD20, CD22, CD24, and CD79a. For these patients, karyotype analysis shows frequent alterations such as hyperploidy or t(12;21)(p13;q22), i.e., associated with a favorable prognosis. Cytogenetic alterations with poor prognosis are t(19;22)(q34;q11.2), t(1;19)(q23;p13.3) or t(4;11)(q21;q23) translocations (7–10).

For these malignancies, the prognosis in pediatric patients is excellent with 90–95% of cases achieving complete remission after chemotherapy. First-line chemotherapy for patients with ALL younger than 65 years is the intensive chemotherapy like Hoelzer protocol or hyper-CVAD (11). In patients with Ph^+^ALL, chemotherapy in combination with tyrosine kinase inhibitors (TKIs) is preferred (12). Ph^+^ALL patients were considered to be at high risk for disease progression or relapse before TKIs were introduced. However, the clinical outcome of patients with Ph^+^ALL has been significantly improved with the addition of TKIs (13). For patients with relapsed ALLs younger than 65 years, the salvage chemotherapy regimen is not well established (14, 15). Ph^+^ ALLs can be treated with a protocol in combination of chemotherapy with TKI (16). For older patients, with comorbidities, therapy consists of 600–800 mg/day of imatinib plus 1 g/kg prednisone for 30 days and continues with the administration of 600–800 mg imatinib on the long term. For older patients, induction chemotherapy includes the administration of vincristine plus doxorubicin, dexamethasone, or prednisone and intrathecal administration of 15 mg methotrexate at day 1 (17). As maintenance therapy, patients receive a less-intensive continuation regimen.

Allogeneic stem cell transplantation (SCT) is indicated in B-cell ALL for the patients with a second complete remission, after the failure of the first-line chemotherapy. Patients may undergo up-front allogeneic SCT in complete remission if they have unfavorable prognostic factors (18, 19), including positive minimal residual disease (MRD). The term MRD represents the low-level disease, which persists and is characterized by the presence of a few malignant phenotype bearing cells not detectable by morphologic criteria (20). MRD has an incredibly important prognostic value in hematological malignancies, with laboratory protocols evolving into routing follow-up of patients who undergo chemotherapy. Such tools include multiparametric flow cytometry and quantitative PCR of Ig (19, 21). Flow cytometry is used to detect the aberrant immunophenotypes of malignant cells, but this method is pushed at limits not present in all laboratories. The role of detecting MRD is to help in taking therapeutic decisions regarding continuing or not a treatment taking into consideration the number or malignant cells that remained in that patient and that can cause relapse (22, 23).

In patients with relapsed/refractory Ph-negative B-ALL, blinatumomab can be an option. Blinatumumab is a byspecific antibody that targets both CD3 and CD19, and it has a reported complete remission rate of 67%, but it is also associated with adverse effects such as cytokine release syndrome (CRS), high fever, nausea, headaches, and hepatic and neurologic side effects (24–27). For Ph-negative ALL, in August 2017, the Food and Drug Administration (FDA) approved the use of inotuzumab ozogamicin (28, 29), a monoclonal antibody anti-CD22, but also chimeric antigen receptor (CAR) T-cells (30).

## CAR T-Cells in Cancer Immunotherapy

**Figure 1:**
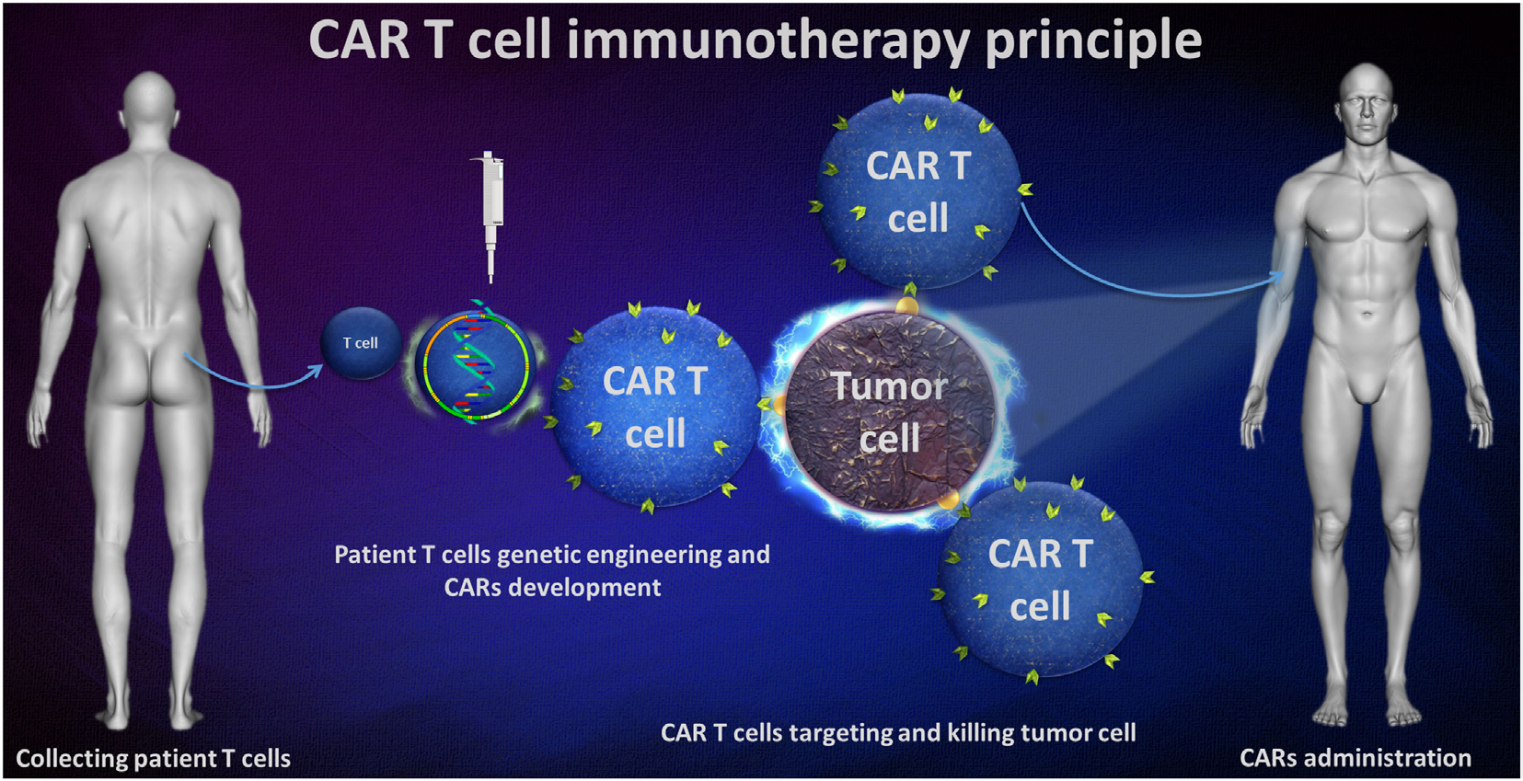
Chimeric antigen receptor (CAR) T-cell immunotherapy principle.

The principle of this immunotherapy involves genetic engineering on patient T-cells to express a surface receptor for direct targeting the tumor cells. Bu using a recombinant technology, a T-cell receptor (TCR) construct was developed, formed by tumor associate antigen-specific single-chain variable fragment (scFv) antibody that is fused with a transmembrane domain (TMD) and then with a intracellular T-cell signaling domain. All these processes leaded to a CAR capable of specific tumor cell binding and activating T-cells to achieve cytotoxic potential (Figure 1).

Chimeric antigen receptor T-cell therapy emerges with various advantages, both from a basic science point of view as well as for clinicians. First, CAR T-cells act independent of the HLA phenotypes, becoming useful for different patients, no matter if their cells express it or not because some tumors tend to downregulate it. The therapy acts in both CD4^+^ and CD8^+^ cells and allows for additional T helper and T cytotoxic cell action.

Chimeric antigen receptor T-cell technology has seen a rapid development over the last decade following the potential that these cells have in treating malignant diseases (31–33). The use of animal models in the preclinical assessment of CAR T-cells makes the molecular and genetic mechanisms of these cells interacting with a certain pathological process accessible to research and experiments, which would otherwise be obviously impossible in humans due to ethical reasons. Thus, the ethical justification of developing such animal models would be to have an experimental approach in extending the knowledge of CAR T-cells. By using these animal models, we have to assess not only the potential that these modified cells would have as a therapeutic approach in various malignancies but also the potential toxic effects that these cells may have on healthy organs.

It is a generally accepted principle that very few therapeutic compounds deliver a clinical response without toxicity, and studies have shown that CAR T-cells are not an exception to this rule (34). Still, a harm–benefit analysis that would be in favor of a positive clinical outcome would sway the balance in the potential use of these cells in a clinical setting. While classical chemotherapy is related with non-selective cytotoxicity such as mucositis and myelosuppression, CAR T-cell therapy (CAR-T) is related with a specific type of toxicity expressed as immune-mediated adverse effects. Some authors have reported the fact that these adverse effects may persist in the long term, extending the duration of possible toxicities beyond that of conventional pharmaceutical molecules (34, 35), as transplanted CAR T-cells have been shown to survive up to 6 months in human patients (36), after having been expanded *ex vivo* for up to 56 days with partially insufficient costimulation (37).

One of the first documented adverse effects of CAR-T in clinical use is the CRS and B-cell aplasia (38–42). CRS is caused mainly by the expansion of the infused T-cells (43), but other cells including B, T, and natural killer (NK) cells, and myeloid cells such as dendritic cells, monocytes, and macrophages seems to contribute to the development of CRS. All of these cells become active and release *en-masse* inflammatory cytokines, thus the paraclinical hallmark of this syndrome being elevated levels of these cytokines. The current concepts in the diagnosis and management of CRS classifies the clinical and laboratory parameter range from mild CRS, expressing constitutional symptoms and grade 2 organ toxicity, to severe CRS, which shows a grade 3 organ toxicity and prompts the need for an aggressive clinical intervention because of the potentially life-threatening toxicity (41, 44). CRS usually occurs around 6 days after the cells are transplanted (41, 45–47). Organ damage appears when engineered T-cells cross-react with a normal tissue or cells that has similar antigen expression with the malignant one (48, 49). The timing between CAR T-cell infusion and CRS is linked often to the release of inflammatory cytokines such as interleukin (IL)-6, interferon-gamma, tumor necrosis factor (TNF), IL-2, IL-10, or IL-8. The increased concentration of these molecules is linked to clinical symptoms such as fever or arterial hypotension. Blood tests will show at this point a peak ferritin level, a peak C-reactive protein level, and significant expansion of CAR T-cells, as detected by flow cytometry (50–53).

Cytokine release syndrome may be linked to the activity of CAR T-cells taking into consideration the immunological cascade following the T-cell activation mediated by the CARs in opposition to native TCR activation, with important clinical consequences, as stressed out by Singh et al. (54). Furthermore, Teachey et al. (55) present the cases of patients treated at the University of Pennsylvania who have died with a diagnosis of CRS and provided a detailed cytokine profile, concluding that cytokine dynamics is similar to the dynamics of hemophagocytic lymphohistiocytosis. The systemic inflammation is driven by macrophage activation and by elevation in IL-6. A fast and efficient of CRS resolution by IL-6 blockade is achieved by the administration of tocilizumab, a drug used primarily for the treatment of juvenile idiopatic arthritis, according to the FDA. It is also approved in Japan for the treatment of Castleman’s disease and is dosed for CRS every 2–4 weeks, being self-limited and without requiring extended treatment.

Hypogammaglobulinemia is often accompanied with a history of recurrent infections, with the site of infection providing clues to the significance and the type of immune deficiency, as well as to the type of microorganism. Infections often affect both the upper and lower respiratory tracts (sinopulmonary infections, sinusitis, bronchitis/bronchiectasis, or pneumonia) or the gastrointestinal tract (parasitic or bacterial gastroenteritis), as well as infections of the joints or skin. Less common symptoms include septicemia or osteomyelitis (56–58). Immunoglobulin replacement therapy should be considered in patients with severe hypogammaglobulinemia as in primary immunodeficiency (59, 60).

Taking into consideration all aspects in CAR T-cell design and experimental assessment, in this article, we aim to describe the main aspects in CAR T-cell use in ALL, from their molecular structure, to preclinical models and assessment in clinical trials.

## Design of CAR T-Cells

Upon harvesting from patient’s peripheral blood and enrichment, primary T-cells need to be genetically modified for CAR T-cell production (61). A gold standard for transduction of primary T-cells is represented by lentiviral vectors (62–67), as a safer alternative to the retroviral vectors, which pose a genotoxic effect on the genome (68). By their integrative capacity into the host genome, expression of CAR transgenic construct can persist independent of cell division. This characteristic makes lentiviral vectors attractive tools for engineering of CAR T-cells. To limit their natural tropism toward CD4^+^ expressing cells, such as T helper lymphocytes, the lentiviral capsid has subjected to extensive pseudotyping with other viral glycoproteins. Toward this end, vesicular stomatitis virus gp (VSV-G) is the most commonly used glycoprotein for pseudotyping of lentiviral vectors, which offers a broad spectrum of infectivity for different cell types (69–71). However, transduction of quiescent cells, such as B-cells and T lymphocytes, are not permissive to transduction with VSV-G pseudotyped lentiviral vectors; therefore, new glycoproteins have been investigated toward this end. Measles virus (MV) hemagglutinin (H) and fusion (F) glycoproteins have shown promise for pseudotyping of lentiviral vectors, displaying an affinity for quiescent T- and B-cells (72, 73). The drawback of MV-pseudotyped lentiviral vectors is the low titer of virus when compared to the more commonly used VSV-G-pseudotyped lentiviral vectors. Using anion-exchange chromatography might offer an advantage over conventional methods for concentration of viral particles from cellular supernatant, reducing the volume of virus needed for transduction of quiescent lymphocytes (72). Recently, mRNA transfer technologies by electroporation and by endocytosis are proposed as new viral toxicity-free methods, which may also allow the expression of CAR in short term, thus resulting in suppressing cross-reactivity (74).

Although CAR-T has shown promise in clinical setups, there are several limitations that arise from a rapid expansion of the CAR T-cells *in vivo*, which can result in severe cytokine release upon antigen recognition (75). In addition, for anti-CD19 CAR T-cells, the inability to discriminate malignant from normal B-cells can lead to long-term B-cell aplasia (53). Therefore, controlling the function of CAR T-cells would be highly desirable for reducing these potential life-threatening adverse effects. In this regard, researchers have used antibody-based switch molecules that intermediate the immunological synapse between CAR T-cells and malignant cells, leading to a regulated cytotoxic activity (66, 67). Engineered T-cells represent modern-day therapeutic options for cellular therapies, with improved safety and versatility when compared to classic cell therapies, such as SCT. Switch molecules are peptide-engrafted antibody-based molecules that could be used to titrate the therapy to minimize organ toxicity that appears as a result of shared antigen expression with normal tissues. A switch activates a CAR T-cell when it is triggered by both rimiducid and the targeted antigen expressed on the surface of leukemia cells. Current generation CAR T-cell constructs consist of a CD3-ζ domain and one or more costimulatory molecules that are both activated when a cancer antigen binds to the portion of the CAR on the surface of the engineered T-cell. This reliance on antigen for activation of the CAR T-cell results in an unpredictable and inherently uncontrollable therapeutic effect. CAR T-cells that target CD19 have been shown to proliferate in excess of 2.200- to 2.500-fold *ex vivo* expansion in some patients, ultimately comprising over 50% of circulating lymphocytes (76). CAR T-cells for solid tumor, on the other hand, often fail to proliferate or persist at all for more than a few days or weeks and have been largely ineffective (77–80). In normal physiology, conventional T-cells recognize single antigens, but a CAR can be modified to recognize multiple surface antigens, as is the case of universal ectodomain CARs, which incorporate either avidin or a FITC-specific scFvs and recognize malignant cells with multiple antigens (66, 81–83). This is the case of solid tumor antigens targeted by CARs and include CD171, folate receptor α, human growth factor receptor 2 (Her2/neu), carcinoembryonic antigen, or the vascular endothelial growth factor receptor 2 (84). The physician has no effective way to intervene to achieve greater consistency once the cells have been administered. The switch molecule technology is designed to separate the dual costimulatory domain, MC, from the antigen recognition domain and moves it onto a separate molecular switch that rimiducid can control. This separation is designed to control the degree of activation of the CAR T-cells through adjustments to the schedule of rimiducid administration, but still in a tumor-dependent manner. This additional control system allows the engineered T lymphocyte to be “turned off” after the disease remission and tumor elimination with negative MRD. Thus, theoretically, healthy B-cells may repopulate the bone marrow of the leukemia or lymphoma patient. In addition, this strategy can offer an increased specificity for malignant cells, which can be extended for solid tumors as well, as presented in Table 1.

**Table 1:** Current clinical trials regarding CAR T-cell therapy.

Type of CAR T-cell	Targeted tumor antigen	Characteristics	Phase	Coordinating institution	Reference
CD3-ζ domain and one or more costimulatory molecules	CD19	Proliferate in excess of 2.200- to 2.500-fold *ex vivo* expansion in some patients, ultimately comprising over 50% of circulating lymphocyte	Phase I clinical trial	MD Anderson Cancer Center, Houston, USA	(76)

Control of CAR T-cell activity	Switchable CAR T-cell	Dimerizing small molecules	Preclinical research	Cellectis, New York, USA	(85)
				University of California in San Francisco, USA	(86)

	Suicide gene	iCasp9	Phase I clinical trial	Baylor College of Medicine, Houston, USA	(87)
		Antibody-mediated depletion	Phase I clinical trial	Fred Hutchinson Cancer Center, Seattle, USA	(88)

Masked CAR T-cell		Enhance selectivity of CAR T-cells	Preclinical research	CytomX Therepeutics, San Francisco, USA	(89)

Enhance of activity	scFv	Enhance selectivity of CAR T-cells	Preclinical research	MD Anderson Cancer Center, Houston, USA	(90)

Combinatorial antigen targeting		SynNotch CAR circulation	Preclinical research	University of California in San Francisco	(91)

		iCAR	Preclinical research	Memorial Sloan Kettering Cancer Center, New York, USA	(92)

*CAR, chimeric antigen receptor; scFv, single-chain variable fragment*.

Another safety concern is related to insertional mutagenesis potential of integrating vectors. Although an important step was made by switching from oncoretroviral vectors to lentiviral vectors, which are considered as a safer alternative to the former ones due to a relative random insertional pattern. However, the oncogenic potential of lentiviral vectors has been previously reported (93, 94), and this might raise safety issues regarding the use of integrating vectors. Toward this end, efforts have been made to reduce the insertional mutagenesis potential of delivery vectors for CAR into T-cells. Generation of integration-deficient lentiviral vectors and inclusion of a scaffold/matrix-associated region (S/MAR) in the vector backbone displayed comparable cytotoxic effect of CAR T-cells engineered with non-integrating vectors to those that have the integration function unaffected (95). Non-integrating vectors due to the presence of S/MAR element in their design are maintained in subsequent cell generation as an episome.

An alternative to lentiviral vectors could be represented by transposons, as they have been described as efficient gene delivery vectors and has been used for gene therapy applications in clinical trials (96–98). DNA transposons have been used as gene delivery vehicles instead of retrotransposons because their genomic insertions have not been associated with any human disease (99). However, delivery of the transgene is mediated by an encoding transposase that must be provided in trans from the same construct or a second construct, and this might add an extra level of complexity to the experimental setup.

Yet, another alternative to both viral and non-viral delivery could be represented by the newly described gene editing tool, named CRISPR/Cas9 (100, 101). This technology offers the possibility to target virtually any genomic site in a RNA-guided manner. The editing complex futures the Cas9 nuclease and a guide RNA, composed of a CRISPR RNA (crRNA) and a trans-acting crRNA. Upon hybridization of the crRNA to the target sequence, Cas9 generates a double-strand break, which can be repaired by non-homologous end joining, an event that can result in a loss of function of the genomic locus. In the presence of a donor DNA, by a mechanism of homology-directed recombination, an exogenous sequence can be introduced into the targeted locus (102, 103). This knock-in capability of CRISPR/Cas9 can be exploited to deliver CAR expression cassette in a desired genomic locus that does not interfere with gene function and therefore minimizing the genotoxic effects experienced with integrating viral vectors. Recent improvements in gRNA and Cas9 have reduced the off-target effects to a minimum, increasing the chances of CRISPR/Cas9 to reach clinical applicability. Up to date, CRISPR/Cas9 already proved its applicability in the field of immunotherapy by enhancing CAR T-cells potency by knockout diverse genes to improve target recognition and cytotoxic activity (104). This technology can be used to knockout PD-1 or the endogenous TCR in NY-ESO-1 TCR transduced T-cells (104, 105). Therefore, CRISPR/Cas9 will surely make a difference in advancing immunotherapies for malignant disorders, in both hematological and solid cancers. However, further improvements in delivery systems are still to be made, and as stated above, designing more specific and regulated systems are desirable to achieve a controlled activity of CAR T-cells. Still, interesting and exciting features of CAR T-cells have been described by Kawalekar et al. (106), who have concluded that 4-1BB CAR but not CD28 CAR induce memory formation and enhance mitochondrial function and antitumoral potential. Additional functions of CAR T-cells enhance their efficacy as strong persistence is achieved by FAS signal inhibition (107). Gattinoni et al. even suggest that early memory T-cell subsets are suitable candidate for CAR T-cell-based therapy (108).

## Classical CAR Constructs

**Figure 2:**
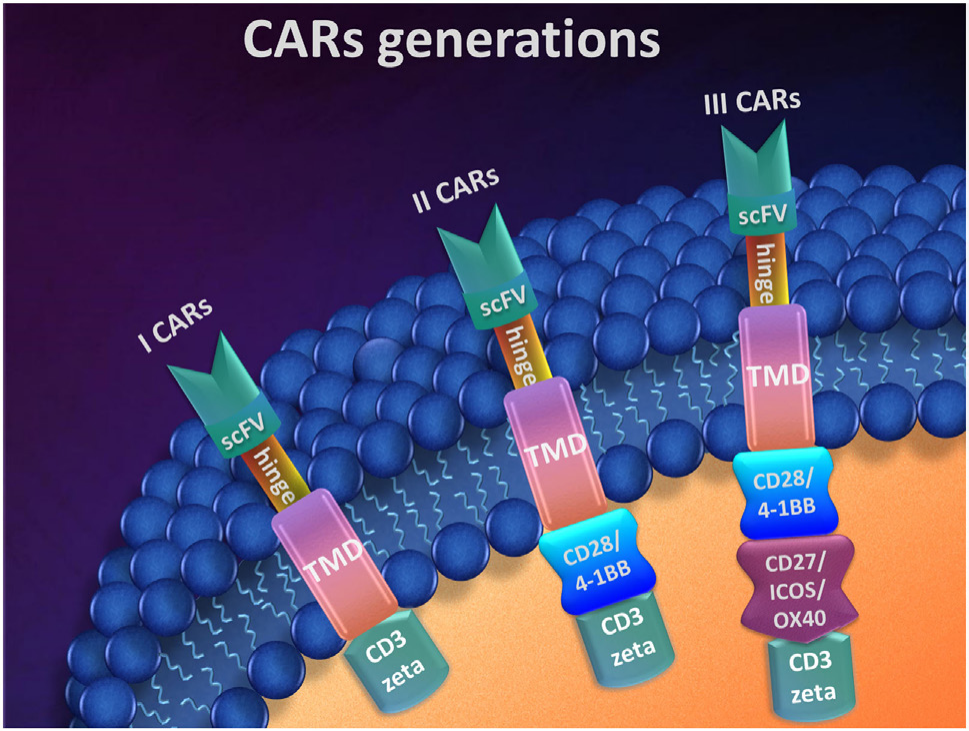
Chimeric antigen receptors (CARs) generations.

The design of CARs can vary, and currently, there are three generations of CARs (Figure 2). The main components of a CAR system are the CD3 zeta intracellular domain of the TCR, the TMD, the hinge, and a scFv. As a result of this composition, the CAR can be defined as a hybrid antigen receptor (109). This basic system describes the first generation of CARs.

The design of the scFv influences the efficiency and the specificity of CAR T-cells in targeting malignant cells. In most cases, it has a murine origin, which determines the anti-CAR immune responses. In spite of their specificity for tumor antigens, in some cases, CAR T-cells target and kill normal cells, resulting in B-cell aplasia, loss of immunity, and finally long-term effects on the patient’s health that may eventually lead to death (58, 110–112). To minimize these drawbacks, further more sophisticated designs of CARs must be developed.

In the design of CARs, key elements are the connection components such as the hinge (also called spacer) and TMD. They form a bound between scFvs and the intracellular domain and are responsible for CARs position and attachment in T-cell membrane. Besides this structural role in CARs design, the hinge morphology characteristics, such as their length and sequence are important for an efficient targeting. The intracellular domain acts as signal transducer. It was shown that the cytoplasmic segment of CD3 zeta plays the principal role due to different functions in activated T-cells and the resting ones. However, this cytoplasmic part cannot activate the resting T lymphocytes. This cytoplasmic part cannot activate the resting T lymphocytes (113). Although the first-generation CARs showed promising results *in vitro* (114–116), these data emphasized the need of at least secondary signal for the fully activation of T-cells. To overcome these limitations, second- and third-generation CARs were developed. By using a costimulatory domain, T-cell activation was achieved *in vitro*, with good persistence *in vivo* (117, 118). Also, incorporation of more potent costimulatory domains enhances T-cells functions *in vivo*, as shown by later studies (119–121).

Furthermore, the absence of highly specific proteins on the surface of some tumor cells is not a drawback for this therapy because CARs can have different modifications (first generation versus third generation) to increase its antitumor activity and are very specific for cell surface molecules (122–124). These receptors recognize and bind different structures, from protein epitopes to glycolipids and carbohydrates (125).

**Figure 3:**
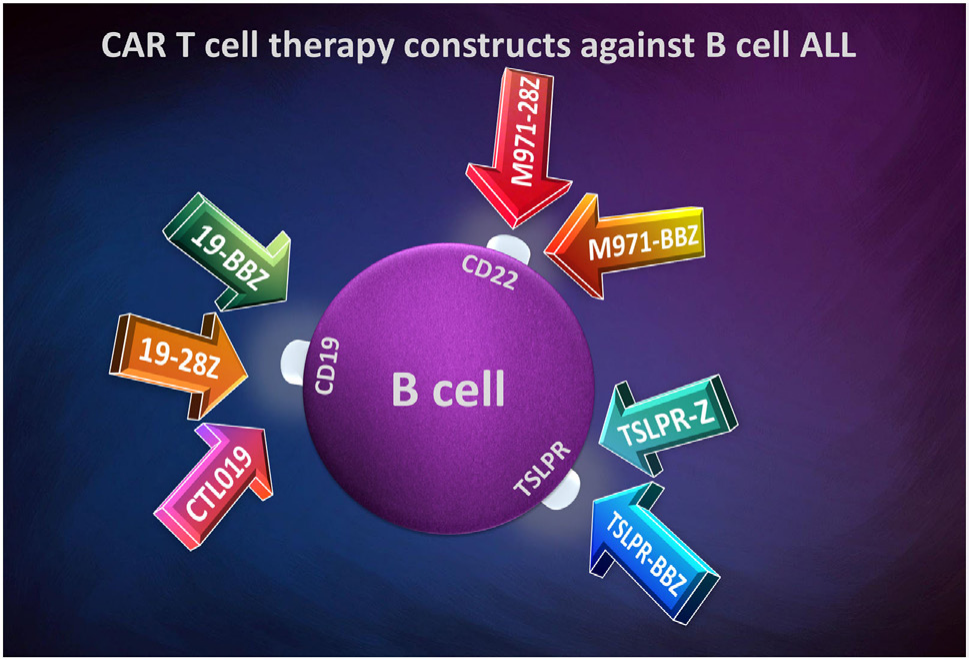
Chimeric antigen receptor T-cell therapy constructs against B-cell acute lymphoblastic leukemia.

An optimal target for CAR T-cells would be a tumor-specific antigen. This would be ideal, but in ALL, no tumor-specific antigens are targeted efficiently. The main difference between a normal and leukemia cell is the antigen expression abundance, increased in the malignant one. In B-cell ALL, it is more efficient to develop CAR constructs depending on B-cell lineage, as shown in Figure 3.

By adding an additional costimulatory signaling domain such as CD28 or 4-1BB at the CD3 zeta intracellular signaling domain, one can build second-generation CARs. Both CD28 and 4-1BB (CD137) costimulatory signaling domains are related with clonal expansion of activated T-cells, being recognized to be related to longtime survival of activated T-cells than CD28 signals (50, 126–128). By further adding another two or more costimulatory signaling domains like CD27, CD28, 4-1BB, ICOS, or OX40 at the CD3 zeta intracellular signaling domain, one can build third-generation CARs (81, 129). CD27, ICOS, and OX40 (CD134) costimulation with CD28 or 4-1BB improves T-cell survival (130–132).

Costimulatory signaling from CD28 is associated with improved CAR T-cell expansion and persistence after their infusion into the patient’s blood stream, with excellent results in indolent B-cell malignancies, as well in ALL. CD28 costimulation is usually provided by antigen-presenting cells and are linked to various signaling molecules of the TNF receptor family such as OX40 and 4-1BB (20). CD28-containing CARs were investigated in a phase I clinical trial in which eight chronic lymphocytic leukemia (CLL) and one ALL patients were recruited (24). Although eight of the patients tolerated the CAR infusion well, one of them had a rapid clinical deterioration and died 48 h following the infusion. Others developed fever with or without arterial hypotension. One of the CLL patients had a partial response, and none of them had B-cell aplasia. The ALL patient was treated in remission, developed B-cell aplasia even if the other hematopoietic series were recovered, a clinical status that lasted until he received an allogeneic SCT after 8 weeks. For this trial, the persistence of the infused CARs was inversely proportional with the tumor burden, being enhanced by prior cyclophosphamide administration. This is an additional reason for the support of lymphodepletion chemotherapy before CAR infusion.

## Second-Generation CAR Constructs for Hematological Malignancies

In present-day clinical trials for hematological malignancies, the most widely used CARs for immunotherapy are the second-generation CARs because of their enhanced viability and efficiency *in vivo* (74, 133). Even if these CARs show a therapeutic potential in the treatment for ALL, severe side effects are a major drawback including CRS and B-cell aplasia (116, 134). Current state-of-the-art CAR design aims at programming T-cells using suicide genes transfer in CAR T-cell constructs (135).

Most of targeting immunotherapies involving CAR T-cell used in B-cell ALL are against the B-cell surface protein CD19. This receptor expresses during B-cell development and is specific to a single-cell lineage. Moreover, this antigen is expressed in almost all B-cell malignancies such as B-cell chronic lymphoblastic leukemia, B-cell ALL, and non-Hodgkin lymphomas. Immunotherapy based on CARs is at its beginnings and various medical centers throughout the world have begun to assess their efficacy both in preclinical setting and in phase I/II clinical trials. Still, sometimes because of different manufacturing and delivery of CARs between various centers, it is somewhat difficult to compare the results obtained between various researchers. This has lead to outcome differences due to the costimulatory sequence in the CAR construct. One such example is the optimization of CD19 CAR T-cell immunotherapy in multicentric clinical trials because of the lack of uniformity of the infused cellular product.

Anti-CD19 CAR T-cell products may vary depending on the institutional design, doses, and T-cell activation and transduction methods. Imai et al. have developed a second-generation CARs, anti-CD19-BB-zeta, by combining an anti-CD19 scFv, a hinge, a CD8 alpha TMD, a CD3 zeta-signaling domain with a 4-1BB co-signaling domain for B-cell ALL therapy. This CAR construct presented a durable expression due to the retroviral transduction mechanism and an efficient cytotoxic activity against ALL cells (136, 137). Another example using the 4-1BB co-signaling domain for CD19^+^ ALL cells is the CTL019 construct, which was obtained using a lentiviral vector. CAR T-cells with this construct were administrated for B-cell ALL patients and showed an increased expansion *in vivo* more than 1,000 times in comparison to the initial dose injected. Good results are reported as a 90% complete remission rate in 30 patients with relapsed and refractory B-cell ALL (110, 124). CAR-based therapy that involves the 4-1BB co-signaling domain can be of high clinical potential for relapsed Ph-positive ALL patients, as shown by Zhu et al. (138). A negative aspect of this therapy refers to the side effects as described above: B-cell aplasia and CRS (124, 139), as well as encephalopathy. Neurological adverse effects have been associated with CAR T-cell activity, all of them being simultaneous or after the CRS. Such side effects include aphasia, confusion, hallucinations, delirium, or even seizures and are thought to be related to CD19 (140–142). Neurological complications following CAR T-cell infusion is closely related to CRS, a systemic inflammatory response incompletely understood so far (43). Similar effects have been described for blinatumomab in Ph-negative relapsed or refractory B-cell ALL, but the mechanism of action is incompletely understood.

By respecting the CD28 co-signaling domain, the construct was integrated in an additional anti-CD19 second-generation CAR construct named 19-28z. This newly described construct proved good results for relapsed or refractory B-cell ALL patients (143). This therapy was shown to have 88% complete response (CR) rates in16 patients with relapsed or refractory B-cell ALL. Still, side effects such as CRS were reported, with high values of C-reactive protein as an indicator of its severity. Another second-generation CAR construct incorporating CD28 signaling domain proved efficiency for 21 chemotherapy-resistant B precursor ALL patients (70% complete remission rate) without encountering prolonged B-cell aplasia after. In addition, the other side effects such as CRS were reversible (144). CAR T-cell-based therapy represents external stimuli that might be significant on the immune system. Hill et al. have addressed this question and investigated the epidemiology of infections 90 days following CAR T-cell infusion for 133 patients diagnosed with a B-cell malignancy (145). They concluded that the incidence and type of infections are correlated to the patients with B-cell malignancies who have received salvage chemoimmunotherapy. The ones with a greater immunosuppression had a higher risk of infections. Still, life-threatening or rare infections were rare, being present especially in the ones who received lymphodepletion chemotherapy. Thus, efficient strategies to prevent infections must be developed to optimize CAR-T.

In some cases, the responses to an anti-CD19 CAR T-cells immunotherapy are not as good as expected, especially when B-cells lose their receptors expression (146, 147). Therefore, the need to identify other suitable targets on B-cells emerged. Other CAR-Ts focus on CD22 surface receptor. The CD22 antigen is another B-cell antigen family member, in fact a Siglec-family lectin, which is expressed during B-cell development and lost upon the differentiation to plasma cells. The tissue distribution of CD22 antigen is similar to CD19 (148). m971 anti-CD22 monoclonal antibody is a part of the derived second-generation CARs (m971-28z or m971-BBz) and have showed good results in treating B-cell precursor ALL patients (149).

Some B-cell ALL patients overexpress the thymic stromal lymphopoietin receptor (TSLPR) due to rearrangements in order of translocations or deletions on CRLF2 gene, which encodes it (150, 151). This receptor may actually be a new target for developing new CAR constructs. Qin et al. have used in their study two CAR constructs: a first-generation CAR and a second-generation CAR (152). The long one included a CD3 zeta intracellular domain, a 4-1BB co-signaling domain, a CD8 TMD, and the scFvs for TSLPR targeting. The shorter CAR proved a greatest activity than the longer one, even when it was comparatively analyzed with second-generation anti-CD19 and anti-CD22 CAR constructs.

## Preclinical Models of CARs in ALL

One of the first documented adverse reactions on CAR-T in preclinical murine models is CRS. It has been shown in a murine model that CAR T-cell infusion-associated CRS can be prevented through the administration of the kinase inhibitor ibrutinib (42). Another potential side effect of CAR T-cell use is graft-versus-host disease (GVHD). To the present date, GVHD is not a real concern regarding CAR-T side effects (153). Provasi et al., using zinc finger nucleases against the endogenous TCR α and β genes, managed to edit Tg T-cells to express only Willms tumor-1 (WT-1)-specific TCRs in a Hu-PBL-severe combined immunodeficiency (SCID) animal model, a SCID mouse grafted with human peripheral blood lymphocytes, of WT-1^+^ leukemia. The results of the study have shown that the mice receiving edited WT-1^+^ T-cells developed neither leukemia nor GVHD 50 days postleukemic transplantation, while mice receiving un-edited WT-1^+^ T-cells had all succumbed to GVHD and mice that did not receive PBMC all died of leukemia (154). Years later, in two clinical reports, patients who underwent allogeneic SCT also received infusions of anti-CD19 CAR allogeneic T-cells from their initial transplant donors. The first report did not identify any GVHD in any of the 8 transplanted patients (155), while the second report showed that 1 of 20 patients developed a worsening of a pre-existing chronic GVHD (156).

Across the large variety and number of preclinical publications focusing on CAR T-cells, very few of them document toxicity in animal models as it would seem normal with any new compound that has a potential use in a clinical setting. Paradoxically, there are numerous studies reporting the clinical use of CAR T-cells even though their safety has not yet been evaluated extensively *in vivo*. An explanation for this phenomenon could include factors such as the large variety of engineered CAR cells, the differences between mouse and human physiology and T-cell biology and the differences in drug metabolism capacity in each species. An example that would confirm this hypotheses would be the fact that one *in vivo* study involves CAR T-cells targeting the Her2/neu antigen, proving the antineoplastic activity and the biological safety of Her2/neu-specific CAR T-cells in transgenic animals with lymphodepletion (157), yet the clinical trial involving the same engineered cells showed that one of the patients died due to a massive CRS (158). The majority of preclinical studies investigating CAR T-cells have focused on verifying their specificity and potency for antineoplastic activity, the key advantage of CARs *in vivo* being the fact that they possess the ability to redirect T-cell effector function without HLA-restriction. The *in vivo* testing of CARs expresses several drawbacks. First of all, the successful engraftment of T-cells in immunocompromised mice is hard to achieve due to the residual elements of the mouse’s innate immune system; another drawback is the fact that even if the engraftment is successful, most of the mice develop GVHD in long-term studies (more than 60 days) (159), which stuns the research on the long-term effects of CAR T-cells in animal models. CAR T-cells target human antigens, which are restricted to transplanted tumor cells in mice, rendering the assessment of their effects on healthy tissues in mice models hard to achieve (46). The humanized NSG mouse has been an indispensable tool for evaluating short-term CAR T-cell activity *in vivo*. CARs that act against ROR1 for mantle cell lymphoma and CD44v6 for AML and multiple myeloma have been tested in humanized NSG mice extensively (160, 161). Humanized mice have been also used to assess the function and efficacy of costimulatory domains such as CD27, ICOS, CD28, and 4-1BB, due to their potential enhanced efficiency in targeting malignancies and augmenting CARs safety (120, 130). In a humanized animal model, the Hu-PBL-SCID NSG mouse, engineered T-cells showed the ability to destroy a cancer cell line that expressed prostate tumor antigens (80). Modern genetic engineering methods like messenger RNA transduction have been used to generate CAR NK cells and to successful target a non-Hodgkin’s lymphoma in a Hu-PBL-SCID NSG model. The study confirmed that activated expanded peripheral blood NK cell (PBNK) became highly cytolytic, eradicating resistant CD20^+^ B-leukemia/lymphoma after nucleofection with anti-CD20 CAR messenger RNA (162). CAR-modified PBNKs are anti-CD20 CAR-modified expanded NK cells significantly mediate immunotherapy-resistant B-cell malignancies. Moreover, authorities have approved two clinical studies using CAR-expressing NK cells for the treatment of B-lineage ALL. One of the clinical studies (NCT00995137) is aimed to identify the maximum tolerance dose of genetically modified NK cells for patients with relapsed or refractory B-lineage ALL at St. Jude Children’s Research Hospital in Memphis, United States. In this clinical study, allogeneic NK cells were first expanded by co-culture with irradiated K562 cells that were modified to express membrane-bound IL-15 and 4-1BB ligand (K562-mb15-4-1BBL) overexpression of these proteins promotes selective growth of NK cells. The *in vitro* expanded NK cells were then transduced with vectors encoding a signaling receptor that binds to CD19, which is only expressed on B-lineage ALL cells. A similar study (NCT01974479) performed by the National University Hospital in Singapore investigated the persistence and phenotype of redirected NK cells in participants with residual B-lineage ALL after chemotherapy. In that study, donor NK cells were activated and expanded by K562-mb15-4-1BBL cell line combined with IL-2 and then transduced with vectors encoding a signaling receptor targeting CD19 (163, 164).

Even if current animal models for CAR T-cells have a poor predictive nature, these may relate to the biological differences between species, a barrier that could be overcome by developing new humanized mice models. Studies in the last decade have focused mainly on their clinical applications with toxicity being neglected as a main research aim. Most of the clinical studies report toxic effects on these engineered cells, which in turn will cause a stronger will of the researchers to better understand the potential mechanisms of *in vivo* toxicity by developing better animal models to this purpose.

## Genetic Engineered T-Cells for the Immunotherapy of ALL

State-of-the-art therapies for ALL have been developed during last decade, with a special emphasis on the role of immunotherapy and T-cells genetic engineering. The first method regarding T-cells genetic engineering was gene transfer of α and β chain subunits of cloned T-cells receptors specific to tumor antigens. The strategy already succeeded for melanoma (165), but it was shown to have limitations in B-cell malignancies because of their restrictions to HLA phenotypes. CAR-T is an attractive targeted immunotherapy, which has proved its potential as an alternative for curing a lot of blood malignancies, especially B-cell ALL.

The outcomes are sometimes different as the different T-cell subsets are different regarding their ability to eliminate the malignant B lymphocyte, even in xenogenic models of the disease (166). This is of crucial importance in B-cell malignancies that have a variable T-cell subset, as is the case of B-cell ALL (51). The overcoming of this problem was attempted by manufacturing CARs from well-defined CD4^+^ and CD8^+^ subsets (129). Even though the selection of manufacturing is slightly more complex, it allows the infusion of a uniform cellular graft, and thus, it made possible to correlate the CAR dosage and toxicity to disease response properly. In B-cell ALL, CD4^+^ T-cells formulated with either bulk CD8^+^ T-cells or central memory cells have led to almost universal CR rates in both arms of the study. A high CR rate was later confirmed by other groups that used engineered T-cells from bulk T-cell subsets (46, 52, 53).

Grupp et al. (45) describe the use of CARs for B-cell ALL in patients with active disease with multiple relapses after chemotherapy. One of the two patients underwent an unrelated cord blood transplant at the time of CAR infusion with PBMC that were 68% of donor origin, whereas the other patient received autologous cells. Both patients went into remission. The cord blood-transplanted patient had a relapse 8 weeks following the T-cell infusion, with CD19-negative leukemic cells. This suggests that a new leukemic clone emerged and escaped the immune recognition of the CAR. Both patients had a systemic inflammatory syndrome and transient neurological symptoms.

MRD-negative CR is higher, and CAR persistence is longer in B-cell ALL patients who were treated is two clinical trials that used CARs that were different and incorporated 4-1BB-costimulated CARs, when compared to two other trials that incorporated a CD28-mediated costimulation (46, 53). These differences in the CAR construct, which included the scFv, the spacer, or the TMD, were additional to other differences in trial comparison such as the clinical characteristics of the patients, the lymphodepletion chemotherapy, or the schedule of treatments. From all of these differences, the choice of lymphodepletion chemotherapy may have the most profound impact on the transferred T-cell expansion and persistence in the patient. This impact might be due to IL-7 and IL-15, highly used in CAR-based immunotherapy (167, 168). B-cell ALL patients who were treated with cyclophosphamide or fludarabine lymphodepletion before CAR infusion had a good T-cell expansion and persistence *in vivo*, whereas the ones who had received cyclophosphamide with fludarabine had a shorter *in vivo* T-cell expansion.

For patients who undergo an allogeneic SCT, there is a theoretical risk of inducing graft-versus-host-disease by the polyclonal activated T-cells. The alternative approach to replace polyclonal CAR T-cells is the transferring of the CAR into T lymphocytes with a well-defined specificity through their native antigen receptor. Thus, it will exclude the alloreactive cells (169–171). This principle was assessed both in the preclinical setting and in a trial for neuroblastoma, and the patients were treated with both polyclonal T-cells and EBV-specific T-cells genetically modified to express the GD2 neuroblastoma antigen (172, 173). The results were afterward confirmed in B-cell hematological malignancies in a trial that included high-risk CLL, transformed CLL, and ALL. All of the patients had relapsed or were at high risk of relapse after a previous allogeneic SCT (155). The CARs were administered without preinfusion chemotherapy. T lymphocytes were activated and expanded with CMV, EBV, and adenoviral antigens and only afterward transduced with the CD19 CARs, thus resulting cells active against all three types of viruses due to their native TCR. Eight patients were treated and infusions were tolerated, without any systemic inflammation or signs of acute GVHD. The CARs were detected in the peripheral blood of all patients 12 weeks following the infusion, with objective responses achieved in 2 of 8 patients, 1 complete remission and 1 partial remission. Still, both remissions were transient. It is worthwhile to mention that the patients for whom long-term follow-up was possible, no B-cell aplasia was recorded, as well as no agammaglobulinemia, suggesting that allogeneic virus-specific T-cells that express CD19 CAR are well tolerated, efficient against B-cell malignancies up to a certain point.

The tumor microenvironment plays an important role in the initiation and progression of a malignancy, including resistance to chemotherapy (137, 138), as previously shown by our group. Lim and June have addressed this issue, linking CAR-T to targeting the malignant microenvironment as even if engineered T-cells may specifically recognize and target a cancer cell, the microenvironment has a suppressive effect on the CAR T-cells, thus limiting its efficacy (139). Thus, combination therapy with checkpoint inhibitors may provide a viable solution for enhancing the antitumor effect of the CAR T-cells, as it was proven by the team of O’Rourke et al. in solid malignancies (140). A good strategy would be to develop the so-called armored CARs, which also express the very efficient cytokine IL-12, which has a pleiotropic on both innate and adaptive T lymphocytes (141). To further augment the antitumor activity, the fourth-generation CAR that contains a transduction domain to promote production of a T-cell-activating cytokine such as IL-12 (so-called armored CAR T) are currently being researched (174, 175). The choice of the “armor” agent is based on the knowledge of the tumor microenvironment and the roles of other elements of the innate and adaptive immune system. Although there are several variants of armored CAR T-cells under investigation, here, we focus on three unique approaches using IL-12, CD40L, and 4-1BBL. These agents have been shown to further enhance CAR T-cell efficacy and persistence in the face of a hostile tumor microenvironment *via* different mechanisms. Other molecules that could be useful in combination with CARs, which could also be used to remodel the malignant microenvironment, is the synNotch receptor system, which may aid in producing specific secreting payloads in response to the recognition in a target antigen, thus turning the CARs into the so-called pharmacytes (142). The synNotch system is a flexible method of programming cells to find and respond to molecular signals of disease. The highly customizable system, known as synNotch, can be used to deliver therapeutic molecules to a disease site or modulate local immune activity. Roybal et al. have used synNotch to instruct immune cells to carry out specific activities in the presence of their targets, such as delivering therapeutic antibodies to tumors or triggering the release of signaling molecules that can dampen overactive immune responses (176). The synNotch system is an adaptation of a naturally occurring receptor molecule called Notch, which facilitates critical cell-to-cell communications in most organisms. Notch receptors are embedded in cells’ outer membranes, with functional components protruding into both the cell’s interior and its external environment. When the exterior part of a Notch receptor connects with its molecular partner, its interior end is freed from the rest of the molecule and moves to the cell’s nucleus, where it activates specific genes.

Thus, CAR T-cells may potentially represent a new age in cancer immunotherapy, with endless possibilities that must be investigated in hematology and oncology, both in the preclinical setting and in phase I to phase III clinical trials, before being approved as standard of care.

## Conclusion

Chimeric antigen receptor T-cells have been reported to show an exceptional activity against B-cell malignancies, may it be CLL, non-Hodgkin’s lymphomas, or ALL in the preclinical setting, as well as in early clinical trials. Despite the excellent results obtained so far, intriguing questions related to the engineering and their clinical activities have emerged. We still do not know exactly the optimal method for transferring CARs into T-cells, may be it lentiviral or retroviral, or which specific costimulatory domain to use. CARs are also related to severe side effects such as tumor lysis syndrome or release of inflammatory cytokines, as well as their effectiveness in replacing the hematopoietic SCT altogether or being just a bridge to transplant as consolidation therapy. Still, the results of CAR T-cells were far from being imaginable 10 years ago, and numerous trials are expanding these opportunities and answering these questions. Up to this point, we can only state that CAR T-cells represent an interesting option for treating B-cell ALL, which became one of standard of cares in this type of cancer.

## Author Contributions

All authors have contributed to the design of the review and to writing of the manuscript.

## Conflict of Interest Statement

The authors declare that the research was conducted in the absence of any commercial or financial relationships that could be construed as a potential conflict of interest.
